# Genomic Characterization of ESBL- and Carbapenemase-Positive Enterobacteriaceae Co-harboring *mcr-9* in Japan

**DOI:** 10.3389/fmicb.2021.665432

**Published:** 2021-08-24

**Authors:** Akihiro Nakamura, Tatsuya Nakamura, Makoto Niki, Tomokazu Kuchibiro, Isao Nishi, Masaru Komatsu

**Affiliations:** ^1^Department of Clinical Laboratory Science, Faculty of Health Care, Tenri Health Care University, Tenri, Japan; ^2^Faculty of Health Sciences, Kyoto Tachibana University, Kyoto, Japan; ^3^Department of Infection Control and Prevention, Osaka City University Hospital, Osaka, Japan; ^4^Department of Clinical Laboratory, Naga Municipal Hospital, Wakayama, Japan; ^5^Laboratory for Clinical Investigation, Osaka University Hospital, Osaka, Japan

**Keywords:** multidrug-resistant Enterobacteriaceae, colistin, *mcr-9*, whole-genome sequence, antimicrobial resistance

## Abstract

Worldwide spread of Enterobacteriaceae resistant to colistin, a polypeptide antibacterial drug for last-resort treatment of carbapenemase-producing Enterobacteriaceae (CPE) infections, is concerning. This study aimed to elucidate colistin MICs and molecular characteristics of *mcr-1* to *mcr-9* of ESBL-producing *Escherichia coli* (ESBL-Ec) and CPE in Japan and clarify the genomic structure of strains harboring *mcr* genes (especially *mcr-9*). This study included 168 ESBL-Ec and 126 CPE strains isolated at Japanese medical facilities. Colistin susceptibility testing and multiplex PCR targeting *mcr-1* to *mcr-9* were performed for all strains with S1-nuclease pulsed-field gel electrophoresis, Southern blot hybridization, and whole-genome sequencing (WGS) with hybrid assembly performed for *mcr* gene-carrying strains. Two CPE strains showed a MIC ≥ 4 μg/ml in colistin susceptibility testing, with no known resistance mechanism detected. However, PCR conducted on all target strains detected three *mcr-9*-carrying strains showing colistin susceptibility. The *bla*_*CTX–M–62*_-positive *E. coli* THUN648 strain simultaneously carried *bla*_*CTX–M–62*_ and *mcr-9* on a 275-kbp plasmid. Besides, *bla*_*IMP–6*_ + *bla*_*CTX–M–2*_-positive *Klebsiella pneumoniae* THUN262 and *bla*_*GES–24*_-positive *Enterobacter kobei* THUN627 had *mcr-9* encoded on the chromosome. Only THUN627 encoded *qseB/C*, which is suggested to be a regulatory gene for *mcr-9*, downstream of *mcr-9*. However, this strain showed no increased expression of these genes in mRNA quantitative analysis under colistin exposure. Colistin MICs of ESBL-Ec and CPE in Japan were all below 2 μg/ml, which is below the epidemiological cutoff (ECOFF) value (https://eucast.org/) or clinical breakpoint (CB) (CLSI M100-S30) reported for colistin, indicating neither “microbiological” nor “clinical” resistance. Several colistin-susceptible Enterobacteriaceae carrying silent *mcr-9* encoded on plasmids and chromosomes have already spread worldwide along with other antimicrobial resistance genes. However, the mechanism of colistin resistance by *mcr-9* remains unclear.

## Introduction

In recent years, the global spread of drug-resistant bacteria has become a concern, and among these bacteria, multidrug-resistant Enterobacteriaceae are remarkable (Nicolas-Chanoine et al., 2014; Mathers et al., 2015). The emergence of carbapenemase-producing Enterobacteriaceae (CPE) that are resistant to carbapenem antibiotics, which are commonly used against severe infections, is a threat to humanity and is currently on the rise both in Japan and abroad (Nakamura et al., 2016; Ohno et al., 2017, 2020). The emergence of Enterobacteriaceae resistant to colistin, a polypeptide antibacterial drug of last resort for the treatment of CPE infections, is being reported, and its global spread is troubling (Liu et al., 2016).

Colistin has been used since the 1950s as a feed additive to treat Gram-negative bacterial infections and to promote healthy growth of food animals (Kawanishi et al., 2017; Poirel et al., 2017). However, the use of colistin in humans was discontinued due to frequent adverse reactions such as renal dysfunction. However, it was reapproved as a last-resort treatment in response to the global increase in multidrug-resistant Gram-negative bacterial infections (Poirel et al., 2017). Therefore, the World Health Organization has listed colistin as an extremely important antibiotic since 2016 (World Health Organization and Who Advisory Group on Integrated Surveillance of Antimicrobial Resistance, 2017). The longtime use of colistin in food animals is thought to be the cause of colistin resistance. Therefore, Japan banned the market sale of colistin as a feed additive in 2018, and many other countries have also banned colistin for this use due to public health considerations (Shen et al., 2018; The Bureau of Investigative Journalism, 2018; Amr Insights, 2019; Market Research.Com, 2019).

There are two main mechanisms of colistin resistance: one is the acquisition of a plasmid-mediated colistin resistance *mcr* gene, and the other is point mutation or inactivation of chromosomal genes such as PmrAB, PhoPQ, CrrAB, and MgrB (Olaitan et al., 2014; Sato et al., 2018; Mmatli et al., 2020). Mutations in PmrAB, PhoPQ, CrrAB, and MgrB affect the phosphorylation of lipid A in lipopolysaccharides, which leads to colistin resistance. Inactivation of MgrB also affects capsule structure, and various other factors such as efflux hyperexpression and porin downregulation are also involved in colistin resistance (Mmatli et al., 2020). The plasmid-mediated colistin resistance *mcr* gene is transmitted across bacterial species, and its trends need to be monitored closely.

The *mcr* gene was first discovered in China in 2015, and many reports have since been published, mainly in Asia (Liu et al., 2016; Cui et al., 2017; Tada et al., 2017; La et al., 2019; Farzana et al., 2020). Cui et al. (2017) reported that 1.4% of *Salmonella* spp. isolated from human clinical samples in China carried *mcr-1*. According to the report of La et al. (2019), when culture and PCR were used to screen human fecal material for Enterobacteriaceae carrying *mcr-1* in Singapore, 9% of the cultures were positive, indicating the presence of a large number of potential carriers. According to a 2020 report by Farzana et al. (2020), 0.3% of *Klebsiella pneumoniae* were found to carry *mcr-8* in Bangladesh, and transmission of this resistance gene has been a problem since 2017. As for the emergence of strains carrying the *mcr* gene in Japan, Tada et al. (2017) first reported the presence of *Escherichia coli* carrying *mcr-*1 in 2017. Except for a few reports since then, there have been no reports of large-scale surveillance (Tada et al., 2017). Among *mcr* genes, *mcr-9* is a novel colistin resistance gene identified in *Salmonella enterica* serotype Typhimurium as reported by Carroll et al. (2019). Although this is a silent colistin resistance gene that has been present in the gene database since around 2010, it has not received much attention as it does not confer colistin resistance. This Mcr-9 protein shares 33–65% identity with the plasmid-encoded Mcr-1 to -8, which have been reported to be phenotypically colistin resistance enzymes, and is most similar to Mcr-3 (Carroll et al., 2019; Kieffer et al., 2019). It has been reported that the mRNA levels of *mcr-9* increased with exposure to colistin, thus causing the MIC to increase (Carroll et al., 2019). The levels of *mcr-9*-induced expression are related to the presence of *qseB* and *qseC* genes downstream. A significant increase in the mRNA expression level of the *qseB/C* genes was observed with exposure to colistin, suggesting that overexpression of *mcr-9* is associated with the QseC/QseB binary system (Carroll et al., 2019).

The purpose of this study was to elucidate the colistin MICs and molecular characteristics of ESBL-producing *E. coli* (ESBL-Ec) and CPE harboring *mcr-1* to *mcr-9* in Japan and to clarify the genomic structure of strains harboring *mcr* genes.

## Materials and Methods

### Bacterial Isolates

All 126 strains of CPE isolated from 2001 to 2018 at 18 Japanese medical facilities from Western Japan (17 acute-care hospitals and 1 commercial laboratory) and 168 ESBL-Ec strains extracted randomly were included in the study (Table 1). The 126 strains of CPE were provided in the framework of the Study of Bacterial Resistance in the Kinki Region of Japan (SBRK), of which 37 strains were those used in our previous four studies (Ohno et al., 2017, 2020; Yamasaki et al., 2017; Kuchibiro et al., 2018) and 89 strains were used in the present study. Two of the four studies were epidemiological studies of CPE in a primary care hospital in Tenri, Japan, conducted from 2010 to 2015, and these included fecal isolates (Ohno et al., 2017, 2020). Besides, one study conducted an outbreak survey of CPE (*bla*_*GES*_ group) in a primary care hospital in Wakayama City, Japan, in 2009, and the source was blood (Yamasaki et al., 2017). One study was collected for the purpose of epidemiological study of CPE from 2000 to 2016 in 15 facilities in Kinki region, Japan, and these did not include fecal isolates (Kuchibiro et al., 2018). Thirty-seven strains of CPE were randomly selected from these four studies. Besides, 89 strains of CPE were collected for the epidemiological study in SBRK. Moreover, the Association of Japan Community Healthcare Organization (JCHO) hospitals and SBRK provided the 168 strains of ESBL-Ec, all of which were also used in our previous studies (Nakamura et al., 2012, 2016; Shibasaki et al., 2016). One of the three studies were epidemiological studies of ESBL-Ec in a primary care hospital in Tenri, Japan, conducted from 2011 to 2012, and the source is feces (Nakamura et al., 2016). Besides, one study was collected for the purpose of epidemiological study of ESBL-producing Enterobacteriaceae from 2000 to 2009 in 18 facilities in Kinki region, Japan, and these did not include fecal isolates (Nakamura et al., 2012). One study was collected for the purpose of epidemiological study of ESBL-producing Enterobacteriaceae in 2012 in 11 facilities in all Japan, and these did not include fecal isolates (Shibasaki et al., 2016). A total of 168 strains of ESBL-Ec were randomly selected from these three studies. This study was approved by the ethical committee of Tenri Health Care University (approval no. 115). Bacterial species identification except for isolates carrying the *mcr* gene was performed using MALDI Biotyper (Bruker Daltonik, Bremen, Germany), and WGS with hybrid assembly was also used to confirm species identification of *mcr*-positive isolates (KmerFinder 3.2)^1^. Screening of the β-lactamase resistance mechanism and multiplex PCR was performed following previous reports (Nakamura et al., 2016; Ohno et al., 2017; Kuchibiro et al., 2018). Seven carbapenemase gene types (*bla*_*IMP–1*_, *bla*_*IMP–2*_, *bla*_*VIM*_ group, *bla*_*KPC*_ group, *bla*_*GES*_ group, *bla*_*NDM*_ group, and *bla*_*OXA–48*_) and six ESBL gene types (*bla*_*SHV*_ group, *bla*_*TEM*_ group, *bla*_*CTX–M–1*_, *bla*_*CTX–M–2*_, *bla*_*CTX–M–8*_, and *bla*_*CTX–M–9*_) were determined (Nakamura et al., 2016; Kuchibiro et al., 2018; Ohno et al., 2020). In addition, PCR amplicon sequencing (Sanger sequencing) was performed on the entire length of the *bla*_*SHV*_ and *bla*_*TEM*_ groups, and only those belonging to 2be as per the classification by Bush and Jacoby (2010) are listed in Table 1.

**TABLE 1:** Characteristics of carbapenemase-producing Enterobacteriaceae and ESBL-producing *Escherichia coli* used in this study.

Group	Species	β-Lactam-resistant mechanism (ESBL or carbapenemase)
CPE (126)	*Escherichia coli* (65)	*bla*_*IMP–1*_ + *bla*_*CTX–M–2*_ (46) *bla*_*IMP–1*_ + *bla*_*CTX–M–2*_ + *bla*_*CTX–M–9*_ (11) *bla*_*IMP–1*_ (2) *bla*_*IMP–1*_ + *bla*_*CTX–M–2*_ + *bla*_*CTX–M–1*_ (2) *bla*_*NDM*_ group (2) *bla*_*IMP–1*_ + *bla*_*CTX–M–9*_ (1) *bla*_*NDM*_ + *bla*_*CTX–M–9*_ (1)
	*Klebsiella* *pneumoniae* (42)	*bla*_*IMP–1*_ + *bla*_*CTX–M–2*_ (19) *bla*_*IMP–2*_ + *bla*_*CTX–M–1*_ (10) *bla*_*IMP–1*_ (5) *bla*_*IMP–1*_ + *bla*_*CTX–M–1*_ (2) *bla*_*KPC*_ group + *bla*_*CTX–M–9*_ + *bla*_*SHV–12*_ (2) *bla*_*GES*_ group (1) *bla*_*GES*_ group + *bla*_*CTX–M–1*_ (1) *bla*_*KPC*_ group (1) *bla*_*NDM*_ group (1)
	*Citrobacter freundii* (6)	*bla*_*IMP–1*_ + *bla*_*CTX–M–2*_ (5) *bla*_*IMP–1*_ (1)
	*Enterobacter* *cloacaecomplex* (6)	*bla*_*IMP–1*_ (4) *bla*_*IMP–1*_ + *bla*_*GES*_ group (1) *bla*_*GES*_ group (1)
	*Klebsiella oxytoca* (4)	*bla*_*IMP–1*_ + *bla*_*CTX–M–2*_ (4)
	*Klebsiella* *aerogenes* (2)	*bla*_*IMP–1*_ (1) *bla*_*IMP–1*_ + *bla*_*CTX–M–2*_ (1)
	*Citrobacter koseri* (1)	*bla*_*IMP–1*_ + *bla*_*CTX–M–2*_ (1)
ESBL-Ec (168)	*Escherichia coli* (168)	*bla*_*CTX–M–9*_ (85) *bla*_*CTX–M–2*_ (47) *bla*_*CTX–M–1*_ (36)

*CPE, carbapenemase-producing Enterobacteriaceae; ESBL-Ec, extended- spectrum β-lactamase-producing *Escherichia coli.**

### Antimicrobial Susceptibility Testing

The agar dilution method (Sigma-Aldrich, Tokyo, Japan) was implemented using colistin sulfate powder for all strains, based on the Clinical and Laboratory Standards Institute (CLSI) M07 11th edition and M100 30th edition (Clinical and Laboratory Standards Institute, 2018, 2020). We set the measurement range of colistin between 0.03 and 128 μg/ml. The microdilution method and *E*-test (bioMérieux Japan Ltd., Tokyo, Japan) were additionally performed for strains showing a MIC of 4 μg/ml or higher with the agar dilution method. All methods were quality checked using standard bacterial strains: *E. coli* ATCC25922, *Pseudomonas aeruginosa* ATCC27853, and *E. coli* NCTC13846 (*mcr-1* positive).

### Molecular Analysis of Plasmid-Mediated *mcr*-Gene Using Multiplex PCR

All target strains were screened for *mcr-1* to *mcr-9* by conventional PCR, which was performed based on the multiplex-PCR methods reported by Lescat et al. (2018) for *mcr-1* to *mcr-5* and Borowiak et al. (2020) for *mcr-6* to *mcr-9*. DNA was extracted using the Cica Genius DNA extraction test (Kanto Chemical Co., Inc., Tokyo, Japan), and PCR target genes were amplified using GoTaq^®^ Green Master Mix (Promega K.K., Tokyo, Japan). Strains harboring plasmid-mediated *mcr* gene underwent antimicrobial susceptibility testing using the microdilution method and *E*-test.

### S1-Nuclease Pulsed-Field Gel Electrophoresis (S1-PFGE) and Southern Blot Hybridization Using the *mcr* Gene

Bacterial lysates of strains carrying any of the *mcr* genes from *mcr-1* to *mcr-9* that were cultured overnight were enclosed in Seakem^®^ Gold agarose plugs (Lonza Japan Inc., Tokyo, Japan). After agar plugs were prepared, the chromosomal DNA was digested by S1-nuclease (TaKaRa Bio, Shiga, Japan) under an enzyme volume of 5 U and reaction time of 1 h. Migration was performed for 19.5 h using the CHEF-DR-III system (Bio-Rad Laboratories, Inc., Hercules, CA, United States) under a switch time of 5.3–34.9 s and a voltage of 6.0 V/cm. After migration, ethidium bromide staining was performed, followed by imaging with ChemiDoc (Bio-Rad Laboratories, Inc.). Agarose gel obtained by S1-PFGE as mentioned above was transferred overnight using nylon membranes, and Southern blotting was performed with a *mcr* gene-labeled DIG probe (Roche Diagnostics, Inc., Tokyo, Japan) for 20 h at 40°C. The *mcr* coding plasmid was detected by ChemiDoc after chemiluminescence using CDP Star (Roche Diagnostics, Inc.), and the plasmid size was confirmed. In addition, the β-lactamase-producing genes (*bla*_*IMP–6*_, *bla*_*GES*__–24_, and *bla*_*CTX–M*__–62_) of the strains harboring *mcr-9* were also subjected to Southern blotting using DIG probe as described above. The primers used for each were those of Borowiak et al. (2020) for *mcr-9*, Nishio et al. (2004) for *bla*_*IMP–6*_, Weldhagen and Prinsloo (2004) for *bla*_*GES–24*_, and Yagi et al. (1997) for *bla*_*CTX–M–62*_. Southern blotting was performed using the method of Yamasaki et al. (2017).

### WGS and Bioinformatic Analysis

Whole-genome sequencing with a hybrid assembly of strains harboring the plasmid-mediated *mcr* gene and strains with colistin MIC greater than 4 μg/ml was performed using MiSeq (Illumina, Inc., CA, United States) and MinION (Oxford Nanopore Technologies, Oxford, United Kingdom). We used the DNeasy PowerSoil Pro Kit (Qiagen, Hilden, Germany) for DNA extraction. The Nextera DNA sample preparation kit (Illumina, Inc.) was used to tune the library for MiSeq sequencing, and 300-bp paired-end sequencing was performed for the tuned library using Nextera XT Index Kit v2 (Illumina, Inc.) and MiSeq Reagent Kit v3 (Illumina, Inc.). As for MinION sequencing, the library preparation was performed using Ligation Sequencing Kits 1D (Oxford Nanopore Technologies). Long-read sequencing was performed for the tuned library using MinION Flow cell R10.3 (Oxford Nanopore Technologies). The long reads obtained by MinION were assembled using Flye v2.8.1 and Canu v1.7.1, and the Miseq data were used to correct errors in the long-read assemblies using Pilon v1.22.

The complete genome sequencing data obtained by the above methods were checked for the presence of various acquired drug resistance genes, including the *mcr* gene and their localization with ResFinder 4.0 from the Center for Genomic Epidemiology website^2^. BLAST search, comparison to similar sequences using BLAST Ring Image Generator (BRIG), and *mcr* gene peripheral structure analysis by Easyfig were performed for the contig sequences coding the *mcr* gene.

For strains with a MIC of 4 μg/ml or higher in colistin susceptibility testing, the sequences obtained by WGS with a hybrid assembly were searched for staining of colistin chromosomal resistance mechanisms according to previous reports (Sato et al., 2018; Mmatli et al., 2020): *pmrAB*, *phoPQ*, *crrB*, and *mgrB*.

### RT-qPCR of *mcr-9* Gene and *qseB/C* Gene

In strains carrying *mcr-9*, mRNA were assayed for the expression of *mcr-9* and *qseB/C* using the method of Kieffer et al. (2019). The bacterial strains were cultured in LB broth containing 0.25 μg/ml and 1 μg/ml of colistin for 4 or 24 h with shaking. RNA was extracted from the culture solution using an RNA Protect Bacteria Reagent (Qiagen) and RNeasy Mini Kit (Qiagen). A StepOnePlus real-time PCR system (ThermoFisher Scientific K.K., Tokyo, Japan) was used to perform RT-PCR, and PCR target genes were amplified using a Power SYBR Green RNA-to-Ct 1-Step Kit (ThermoFisher Scientific K.K). Each assay was performed in duplicate. Quantitative values were calculated by relative quantitative PCR using *E. coli* ATCC25922 as the reference for the Relative Quantification ratio. We used the GAPDH gene as an internal control (Carey et al., 2008).

## Results

### Susceptibility Testing of Colistin and WGS of Strains With MIC of 4 μg/ml or Higher

**FIGURE 1:**
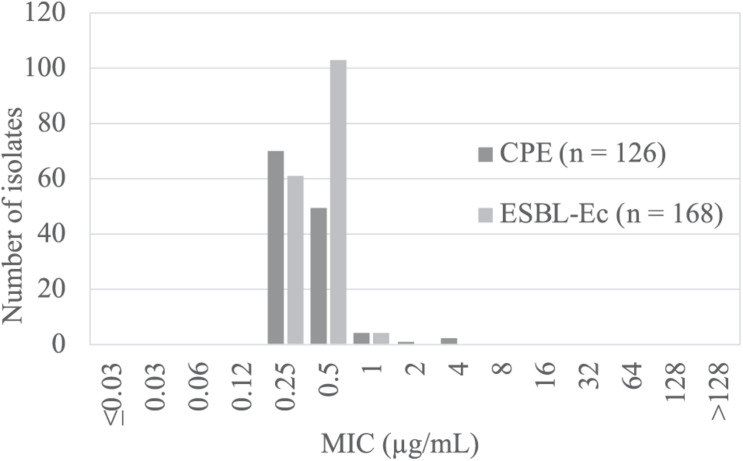
Results of colistin susceptibility testing in carbapenemase- producing Enterobacteriaceae (CPE, dark gray) and ESBL-producing *Escherichia coli* (ESBL-Ec, light gray).

The results of colistin susceptibility testing using the agar dilution method for all target strains are shown in Figure 1. Distribution of the colistin MIC was unimodal, ranging from 0.25 to 4 μg/ml. Two strains, *E. coli* BPML00000000, positive for *bla*_*IMP–6*_ + *bla*_*CTX–M–2*_ + *bla*_*CTX–M–27*_, and *E. coli* BPMM00000000, positive for *bla*_*IMP–1*_, showed MICs above the CLSI breakpoint of 4 μg/ml. Table 2 shows the results of antimicrobial susceptibility testing and WGS of these two strains (GenBank accession numbers: BPML00000000: SAMD00334384 and BPMM00000000: SAMD00334385). The colistin MIC of these two strains were 0.5 μg/ml and 0.5 μg/ml by the microdilution method and 0.25 μg/ml and 0.25 μg/ml by *E*-test, respectively, and they were not carriers of the plasmid-associated *mcr* gene. They were not observed to have the previously reported chromosomal colistin resistance-associated mutations: amino acid substitutions L105P, I128N, and G144S in *pmrA*, amino acid substitutions C84Y, D149Y, L10P, G206D, and *Δ*27–45 in *pmrB*, and amino acid substitution R6H in *phoQ*. In addition, these species were *E. coli*, which also did not harbor *crrB* and *mgrB*.

**TABLE 2:** Genetic characterization of two strains showing MIC of 4 μg/ml using agar dilution method and MICs using microdilution method and *E*-test.

Strain No.	Species	Year of isolation	Region	MLST	Plasmid	Accession number	Acquired antimicrobial resistance gene	MICs of microdilution method																		CL-MICs of *E*-test
								ABPC	A/S	PIPC	CEZ	CTX	CAZ	CPDX	S/C	AZT	IPM	MEPM	FOM	AMK	GM	MINO	LVFX	CPFX	CL	
BPML00000000	*Escherichia* *coli*	2013	Kochi	ST131	IncFIA, FIB, FII, N	SAMD00334384	*aac(6′)-Ib-cr*, *aadA2b*, *tet(A)*, *sul1*, *bla*_*IMP–6*_, *bla*_*CTX–M–2*_, *bla*_*CTX–M–27*_, *mdf(A)*	>32	>8	>64	>16	>32	>32	>32	>32	>16	0.5	16	≤4	≤8	4	≤2	>8	>4	0.5	0.25
BPMM00000000	*Escherichia* *coli*	2014	Kagawa	ST131	IncFIA, FIB, I1-I, A	SAMD00334385	*aph(3″)-Ib*, *aph(6)-Id*, *aadA5*, *mph(A)*, *mdf(A)*, *dfrA17, sul1*, *sul2, tet(A)*, *bla_IMP–1_*	>32	>8	8	>16	>32	>32	>32	32	≤4	1	2	≤4	≤8	≤2	≤2	>8	>4	0.5	0.25

*ABPC, ampicillin; A/S, ampicillin/sulbactum; PIPC, piperacillin; CEZ, cefazoline; CTX, cefotaxime; CAZ, ceftazidime; CPDX, cefpodoxime; S/C, sulbactam-cefoperazone; AZT, aztreonam; IPM, imipenem; MEPM, meropenem; FOM, fosfomycin; AMK, amikacin; GM, gentamycin; MINO, minocycline; LVFX, levofloxacin; CPFX, ciprofloxacin; CL, colistin.*

### Search for *mcr-1* to *mcr-9* in All Target Strains Using Multiplex PCR

Although all strains were negative for *mcr-1* to *mcr-8*, three strains, *K. pneumoniae* THUN262 (*bla*_*IMP–6*_ + *bla*_*CTX–M–2*_-positive) and *Enterobacter kobei* THUN627 (*bla*_*GES–24*_-positive) from the CPE group and *E. coli* THUN648 (*bla*_*CTX–M–62*_-positive) from the ESBL group, were positive for *mcr-9*. The colistin MIC of these three strains using the agar dilution method was 0.5 μg/ml (microdilution method = 0.25 μg/ml, *E*-test = 0.125 μg/ml) for THUN262, 0.25 μg/ml (0.25 μg/ml, 0.125 μg/ml) for THUN627, and 0.5 μg/ml (0.5 μg/ml, 0.125 μg/ml) for THUN648, respectively.

### S1-PFGE and Southern Blot Hybridization Targeting *mcr-9* and Each *bla* Gene

**FIGURE 2:**
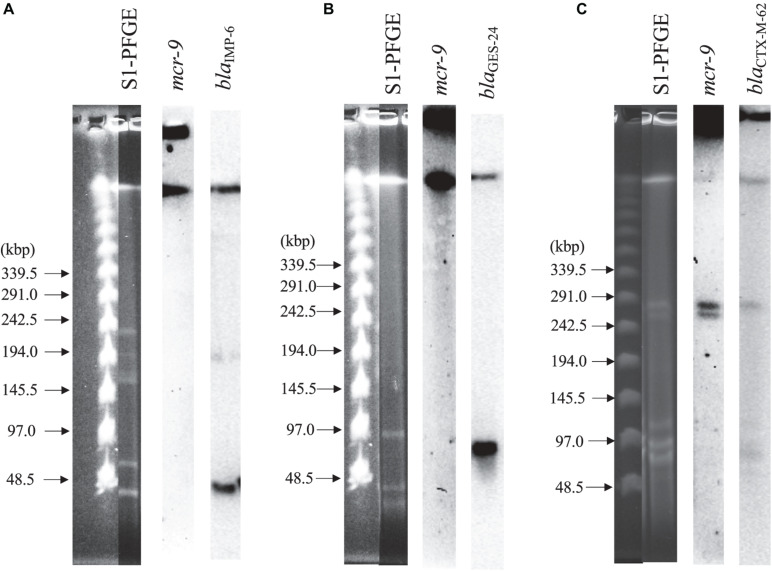
S1-nuclease pulsed-field gel electrophoresis and Southern blot hybridization using three isolates with *mcr-9*. **(A)** *bla*_*IMP–6*_ + *bla*_*CTX–M–2*_-positive *K. pneumoniae* THUN262, **(B)** *bla*_*GES–24*_-positive *E. kobei* THUN627, and **(C)** *bla*_*CTX–M–62*_-positive *E. coli* THUN648.

Figure 2 shows the results of S1-PFGE and Southern blot hybridization for the three strains carrying *mcr-9*. The *mcr-9* of *K. pneumoniae* THUN262 and *E. kobei* THUN627 was not present on the plasmid However, *mcr-9* of *E. coli* THUN648 was encoded on a 260-kbp plasmid harboring *bla*_*CTX–M–62*_.

### WGS and Bioinformatic Analysis of Strains Carrying the *mcr-9* Gene

**FIGURE 3:**
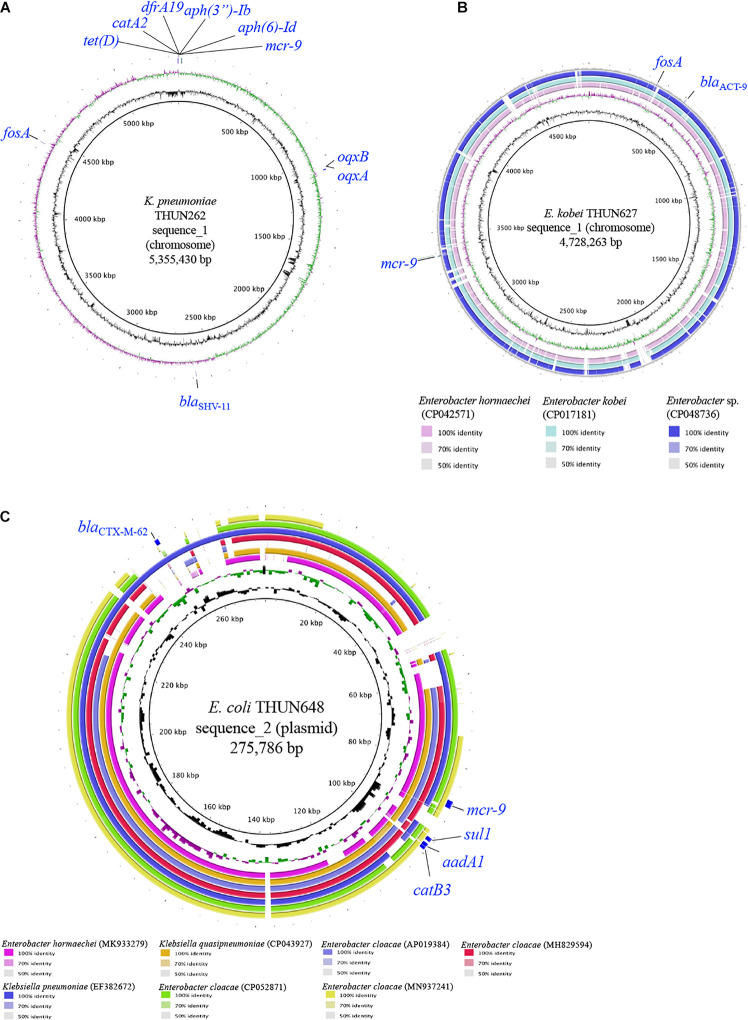
Circular structure of chromosome harboring *mcr-9* of *K. pneumoniae* THUN262 **(A)**, *E. kobei* THUN627 **(B)**, and plasmid harboring *mcr-9* of *E. coli* THUN648 **(C)**. The green and purple circles indicate GC skew, and the black circles indicate GC content.

Table 3 shows the results of WGS and antimicrobial susceptibility testing of the three strains harboring *mcr-9* and the characteristics of each isolate. The genomic sequences of these three strains showed that the *mcr-9* gene was encoded on the chromosome of *K. pneumoniae* THUN262 (GenBank accession number BNSV01000001) and *E. kobei* THUN627 (GenBank accession number BNSW01000001) and on the plasmid of *E. coli* THUN648 (GenBank accession number BNSX01000002). *E. coli* THUN648 with *mcr-9* that was encoded on a 275-kbp plasmid also hosted *bla*_*CTX–M–62*_. Figure 3 shows the results of a BLAST search using the contig sequence that codes *mcr-9* for each bacterial strain and the results of a comparison with similar sequences carrying *mcr-9* using BRIG. No *K. pneumoniae* chromosomal gene data encoding *mcr-9* on the chromosome, such as that on *K. pneumoniae* THUN262, were found in the GenBank database. However, there were three sequences that were most similar to the *E. kobei* THUN627 chromosome sequence: *E. hormaechei* (accession number CP042571) isolated in Australia (75.1% of query coverage and 88.4% of identity), *E. kobei* (accession number CP017181) isolated in Japan (85.6% of query coverage and 99.1% of identity), and *Enterobacter* sp. (accession number CP048736) isolated in China (87.8% of query coverage and 99.2% of identity). In the BLAST search, several sequences similar to the plasmid of *E. coli* THUN648 encoding *mcr-9* and *bla*_*CTX–M–62*_ were found. The most similar sequences were *E. hormaechei*_pMCR-SCNJ07(accession number MK933279) isolated in China (78.8% of query coverage and 99.9% of identity), *K. quasipneumoniae* subsp. *quasipneumoniae*_p17277A_477 (accession number CP043927) isolated in Argentina (53.2% of query coverage and 99.8% of identity), and *E. cloacae* subsp. *cloacae*_pNUH14_ECL028_1 (accession number AP019384) isolated in Japan (69.0% of query coverage and 99.7% of identity). No similar plasmid with *mcr-9* was found in the species of *E. coli* in GenBank. The only virulence factor encoded in this plasmid of *E. coli* THUN648 was *terC*, a tellurium ion resistance protein.

**TABLE 3:** Genetic characterization of three strains with *mcr-9* and MICs using microdilution method and *E*-test.

Strain no.	Species	Year of isolation	Region	MLST	Location	Plasmid type	Accession number	Length	Circular	Acquired antimicrobial resistance gene*^*a*^*	MICs of microdilution method *^*b*^*																		CL-MICs of E-test
											ABPC	A/S	PIPC	CEZ	CTX	CAZ	CPDX	S/C	AZT	IPM	MEPM	FOM	AMK	GM	MINO	LVFX	CPFX	CL	
THU N262	*Klebsiella* *pneumoniae*	2006	Hyogo	ST1 061	Chromo some	–	BNSV01000001	5,355, 430	Yes	*aph(3′′)-Ib, aph(6)-Id*, *bla_SHV–11_*, ***mcr-9***, *fosA, catA2, oqxA*, *oqxB, tet(D), dfrA19*	>32	>8	>64	>16	>32	>32	>32	>32	>16	2	>16	16	≤8	>8	>8	≤0.5	≤1	≤ 1	0.125
					Plas mid	IncHI2, HI2A	BNSV01000003	183, 316	Yes	*aac(6’)-IIc, bla_SHV–11_*, *catA2, sul1*																			
					Plas mid	IncN	BNSV01000004	50, 774	Yes	*aac(6’)-Ib, bla_wIMP–6_*, *bla_CTX–M–2_*, *sul1, tet(A)*																			
					Plas mid	–	BNSV01000006	11, 275	Yes	ND																			
					Plas mid	–	BNSV01000007	8, 740	Yes	ND																			
THUN 627	*Enterobacter* *kobei*	2018	Kyoto	ST 914	Chromo some	*–*	BNSW01000001	4,728, 263	Yes	*bla*_*ACT–9*_, ***mcr-9****, fosA*	>32	>8	>64	>16	8	16	32	>32	≤4	8	8	>128	32	≤2	≤2	1	≤1	≤1	0.125
					Plas mid	IncFIB, FII	BNSW01000002	114,027	Yes	ND																			
					Pla smid	IncN	BNSW01000003	56, 623	Yes	*aph(3”)-Ib, aph(6)-Id*, *qnrS1, sul1, dfrA14*																			
					Plas mid	–	BNSW01000004	38, 839	Yes	*aac(6’)-Ia, cml, sul1*																			
					Plas mid	IncR	BNSW01000005	34, 507	Yes	ND																			
					Plas mid	–	BNSW01000006	1, 845	–	*bla*_*GES–24*_																			
THUN 648	*Escherichia* *coli*	2002	Osaka	ST3 93	Chromo some	*–*	BNSX01000001	5,030, 419	Yes	*bla*_*CTX–M–62*_ *, mdf(A)*	>32	>8	>64	>16	32	4	>32	32	>16	0.5	≤0.12	≤4	≤8	>8	>8	>8	>4	≤1	0.125
					Plasmid	IncHI2, HI2A	BNSX01000002	275,786	Yes	*aadA1, bla*_*CTX–M–62*_, ***mcr-9****, catB3, sul1*																			
					Plas mid	IncFIA, FIB	BNSX01000003	118, 089	Yes	*aadA5, aph(3”)-Ia*, *aph(3”)-Ib, aph(6)-Id*, *bla_TEM–1B_, mph(A)*, *sul1, sul2, tet(B)*, *dfrA17*																			
					Pla smid	–	BNSX01000007	15, 636	Yes	ND																			
					Pla smid	–	BNSX01000010	6, 061	Yes	ND																			

*ABPC, ampicillin; A/S, ampicillin/sulbactum; PIPC, piperacillin; CEZ, cefazoline; CTX, cefotaxime; CAZ, ceftazidime; CPDX, cefpodoxime; S/C, sulbactam-cefoperazone; AZT, aztreonam; IPM, imipenem; MEPM, meropenem; FOM, fosfomycin; AMK, amikacin; GM, gentamycin; MINO, minocycline; LVFX, levofloxacin; CPFX, ciprofloxacin; CL, colistin.*

*^a^Underlined bold text indicates mcr-9 gene.*

**FIGURE 4:**
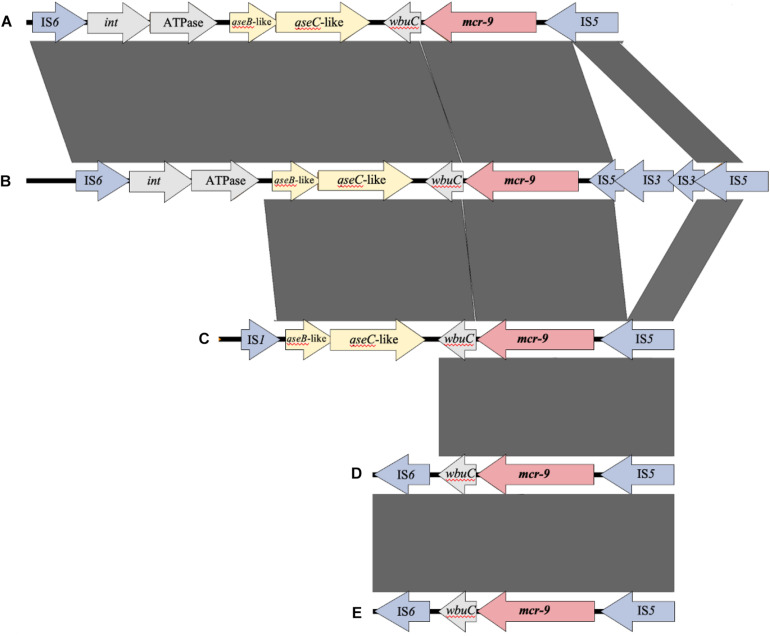
Comparison of the structure around the *mcr-9* gene in each isolate. **(A)** *Leclercia* sp. (accession number CP031102), **(B)** *Enterobacter kobei* THUN627 (this study), **(C)** *Salmonella enterica* (accession number CP029037), **(D)** *Enterobacter cloacae* (accession number CP020529), and **(E)** *Escherichia coli* THUN648 (this study) and *Klebsiella pneumoniae* THUN262 (this study).

Figure 4 shows the results of a comparison of the peripheral structure of *mcr-9* for these three strains using Easyfig. The *qseB/C* gene, which is suggested to be a regulator of *mcr-9* expression (Kieffer et al., 2019), is present downstream of *mcr-9* only in *E. kobei* THUN627, and the other two strains are not carriers of the *qseB/C* gene.

### RT-qPCR of the *mcr-9* and *qseB/C* Genes

**FIGURE 5:**
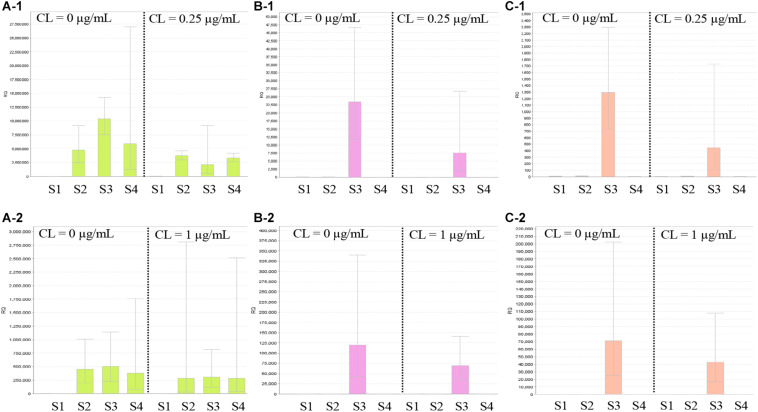
Results of RT-qPCR of *mcr-9* and *qseB/C* genes using three isolates with *mcr-9* and *E. coli* ATCC25922. S1, *E. coli* ATCC25922 without *mcr-9* and *qseB/C*; S2, *K. pneumoniae* THUN262 with *mcr-9*; S3, *E. kobei* THUN627 with *mcr-9* and *qseB/C*; and S4, *E. coli* THUN648 with *mcr-9*. **(A-1)** RT-qPCR of *mcr-9* cultured for 4 h and **(A-2)** for 24 h. **(B-1)** RT-qPCR of *qseB* cultured for 4 h and **(B-2)** for 24 h. **(C-1)** RT-qPCR of *qseC* cultured for 4 h and **(C-2)** for 24 h. Each assay was performed in duplicate. Quantitative values were calculated by relative quantitative PCR using *E. coli* ATCC25922 as the reference for the Relative Quantification ratio.

Figure 5 shows the results of RT-qPCR for the three strains carrying *mcr-9*. The expression of *mcr-9* in all three strains did not increase under colistin exposure or non-exposure and the 4- or 24-h incubation conditions. Also, *E. kobei* THUN627, which has *qseB/C* downstream, did not show an increase in the expression level of *qseB/C*.

## Discussion

The purposes of this study were to elucidate the colistin resistance status of ESBL-Ec and CPE in Japan and to perform a comprehensive search for *mcr-1* to *mcr-9* to elucidate the genome structure that carries the *mcr* gene.

Two strains showed MICs of 4 μg/ml using agar plate dilution based on the CLSI method, and these were carbapenemase-producing strains that were isolated in 2013 and 2014. However, these strains showed susceptibility by the microdilution method and *E*-test. These strains also underwent WGS with hybrid assembly, but no known chromosomal or plasmid resistance mechanisms were detected. Recently, various chromosomal colistin resistance mechanisms have been reported, such as mutation or loss of *pmrAB* and *phoQP*, mutation of *crrB* and presence of *mgrB* (Mmatli et al., 2020). In the present study, the strains that showed a MIC of 4 μg/ml by agar dilution method did not possess any of these known chromosomal colistin resistance mechanisms and may have unknown mechanisms. However, as the microdilution method and *E*-test showed susceptibility, the results may be method dependent. In the future, the mechanisms in these strains will need to be investigated further. Besides, in the present study, three strains carrying *mcr-9* were detected among the bacterial strains with a susceptibility to colistin (MIC of 1 μg/ml or less).

Umeda et al. (2021) isolated *Enterobacter roggenkampii* harboring both IncP6 plasmid coding *bla*_*IMP–1*_ and *bla*_*GES–5*_ and IncHI2 plasmid coding *bla*_*CTX–M–9*_ and *mcr-9* in Osaka, Japan in 2019. These plasmids could be transferred to other strains by conjugation, but *qseBC* was not harbored downstream of *mcr-9*. Besides, Kananizadeh et al. (2020) detected three strains harboring *mcr-9* among 32 *E. cloacae* complex strains showing a MIC ≥ 2 μg/ml collected from 14 hospitals in Japan in 2018. These simultaneously harbored *bla*_*IMP–1*_. In two *E. xiangfangensis*, *mcr-9* was located on a 280/290-kbp plasmid, *qseBC* was not harbored downstream of *mcr-9*, and *bla*_*IMP–1*_ was located on another plasmid (62 kbp). The replicon type of *mcr-9* harboring plasmid was located on IncHI2, as reported by Umeda et al. (2021). Besides, *mcr-9* of one *E. asburiae* was located on the chromosome simultaneously encoded by *bla*_*IMP–1*_ and *bla*_*ACT–9*_, and *qseBC* was encoded downstream of *mcr-9*. The *mcr-9* detected in this study was coded on the chromosome of the two *K. pneumoniae* THUN262 and *E. kobei* THUN627 isolates and on a plasmid of size 276 kbp from *E. coli* THUN648. As mentioned above, the *qseB/C* gene currently recognized as a regulator gene is present only in *E. kobei* THUN627, downstream of *mcr-9*. Of the two strains with *mcr-9* coded on the chromosome, as the chromosome sequence including *mcr-9* similar to that of *K. pneumoniae* THUN262 was not detected in the GenBank database, this chromosome sequence of *K. pneumoniae* THUN262 would appear to be a novel sequence. However, a few chromosome sequences of *E. kobei* THUN627 that included *mcr-9* were present, and strains isolated from various foreign countries besides Japan were also present. The plasmid sequence of *E. coli* THUN648, which had *mcr-9* coded in the plasmid, was observed in multiple bacterial species such as *K. quasipneumoniae* and *E. cloacae* and was also detected in various foreign countries in addition to Japan. This plasmid of *E. coli* THUN648 also harbored *bla*_*CTX–M–62*_ of the ESBL-producing gene. Therefore, it was confirmed that *mcr-9* has already spread worldwide through plasmids along with other antimicrobial resistance genes. In fact, Osei Sekyere et al. (2020) reported that plasmids containing *mcr-9* harbored *bla*_*CTX*__–M–15_ and *bla*_*TEM–1*_.

RT-qPCR for *mcr-9* and *qseB/C* was performed on three strains carrying *mcr-9* after exposure to colistin, followed by relative quantitative comparison. However, no increase in the mRNA expression level was observed in any of the three strains. Although *E. kobei* THUN627 carried the *qseB/C* gene downstream of *mcr-9*, which was suggested to be a regulator gene for *mcr-9* in a previous report (Kieffer et al., 2019), this strain did not show an increase in mRNA expression level. Tyson et al. (2020) examined the colistin susceptibility results of more than 100 strains of *S. enterica* and *E. coli* carrying *mcr-*9 that were isolated in the U.S. All strains were reported to have a MIC of 1 μg/ml or less (Tyson et al., 2020), suggesting that *qseB/C* may not regulate the expression of *mcr-9* by itself.

This study has three limitations. First, the ESBL-producing strains investigated for colistin resistance in this study are limited to *E. coli*. Other Enterobacteriaceae should be examined on a large scale to elucidate the full extent of the *mcr* gene. Second, the resistance mechanism of the two strains that showed a MIC of 4 μg/ml or higher in this study is still not clear. These strains will be investigated further by bioinformatics analysis using WGS. However, in this study, *mcr-9* was found on a plasmid of *E. coli* isolated in 2002 and was found to be on the same plasmid together with the ESBL-producing gene. This suggested that *mcr-9* may have already been spread worldwide together with other antimicrobial resistance genes. Third, screening with agar dilution with a breakpoint of ≥4 μg/ml may have decreased the overall diagnostic sensitivity of the study. In other words, strains with ≤ 2 μg/ml may harbor colistin resistance factors. However, the agar dilution method was reported by Turlej-Rogacka et al. (2018) to result in higher MICs than the broth dilution method. In addition, the colistin agar dilution method is acceptable in CLSI M100-S30. Therefore, we used the agar dilution method to minimize its limitation.

## Conclusion

Strains carrying *mcr-9* on both plasmid and chromosome existed in Japan prior to 2018, when the use of colistin was banned as a feed additive for food animals, and *mcr-9* is already spreading around the world along with other antimicrobial resistance genes. However, the detailed mechanism by which *mcr-9* is involved in colistin resistance is still not clear. We need to pay attention to future trends as *mcr-9* will be highly expressed by various genetic mutations and insertions and has the potential to become a non-silent colistin resistance gene.

## Data Availability Statement

The datasets presented in this study can be found in online repositories. The names of the repository/repositories and accession number(s) can be found in the article/supplementary material.

## Ethics Statement

The studies involving human participants were reviewed and approved by Tenri Health Care University. Written informed consent for participation was not required for this study in accordance with the national legislation and the institutional requirements.

## Author Contributions

AN and MK conceived and designed the experiments. AN, TN, MN, and TK performed the experiments. AN and MK drafted the manuscript. All authors provided critical input and approved the final manuscript.

## Conflict of Interest

The authors declare that the research was conducted in the absence of any commercial or financial relationships that could be construed as a potential conflict of interest.

## Publisher’s Note

All claims expressed in this article are solely those of the authors and do not necessarily represent those of their affiliated organizations, or those of the publisher, the editors and the reviewers. Any product that may be evaluated in this article, or claim that may be made by its manufacturer, is not guaranteed or endorsed by the publisher.

## Footnotes

1
https://cge.cbs.dtu.dk/services/KmerFinder/

2
https://cge.cbs.dtu.dk/services/ResFinder/
